# Tailoring of Novel Bile Salt Stabilized Vesicles for Enhanced Transdermal Delivery of Simvastatin: A New Therapeutic Approach against Inflammation

**DOI:** 10.3390/polym15030677

**Published:** 2023-01-29

**Authors:** El-Sayed Khafagy, Bjad K. Almutairy, Amr Selim Abu Lila

**Affiliations:** 1 Department of Pharmaceutics, College of Pharmacy, Prince Sattam Bin Abdulaziz University, Al-kharj 11942, Saudi Arabia; 2 Department of Pharmaceutics and Industrial Pharmacy, Faculty of Pharmacy, Suez Canal University, Ismailia 41522, Egypt; 3 Department of Pharmaceutics, College of Pharmacy, University of Hail, Hail 81442, Saudi Arabia; 4 Department of Pharmaceutics and Industrial Pharmacy, Faculty of Pharmacy, Zagazig University, Zagazig 44519, Egypt

**Keywords:** anti-inflammatory, bilosomes, rat paw edema, simvastatin, transdermal drug delivery

## Abstract

Simvastatin (SMV), a cholesterol-lowering agent, has antioxidant and anti-inflammatory effects. Nevertheless, the oral use of SMV is linked with poor systemic bioavailability owing to its limited aqueous solubility and extensive first-pass metabolism. The aim of this study was to evaluate the feasibility of transdermal delivery of SMV using bile salt stabilized vesicles (bilosomes) for enhancing the anti-inflammatory potential of SMV. SMV-loaded bilosomes (SMV-BS) were prepared by the thin film hydration technique and optimized by 3^3^ Box–Behnken design. The fabricated SMV-BS were assessed for vesicle size, entrapment efficiency (% EE) and cumulative drug release. The optimized formula was incorporated into HPMC gel and investigated for physical properties, ex vivo permeation, in vivo pharmacokinetic study and anti-inflammatory potential in inflamed paw edema rat model. The optimized SMV-BS showed vesicle size of 172.1 ± 8.1 nm and % EE of 89.2 ± 1.8%. In addition, encapsulating SMV within bilosomal vesicles remarkably sustained drug release over 12 h, compared to plain drug suspension. Furthermore, SMV-loaded bilosomal gel showed a three-fold enhancement in SMV transdermal flux, compared to plain drug suspension. Most importantly, the relative bioavailability of SMV-BS gel was ~2-fold and ~3-fold higher than those of oral SMV suspension and SMV gel, respectively. In carrageenan-induced paw edema model, SMV-BS gel induced a potent anti-inflammatory effect, as evidenced by a remarkable reduction in paw edema, which was comparable to that of the standard anti-inflammatory drug, indomethacin. Collectively, bilosomes might represent a plausible transdermal drug delivery system that could enhance the anti-inflammatory activity of SMV by boosting its skin permeation and its systemic bioavailability.

## 1. Introduction

Inflammation is a natural physiological defensive mechanism of the body against stimuli, infections, cellular stress and cellular damage [1]. Currently, anti-inflammatory drugs are considered one of the most often used drugs globally. Nonetheless, their frequent use is commonly plagued by effectiveness and safety concerns, resulting in highly costly and serious adverse outcomes. As a result, there is an obvious need to develop novel anti-inflammatory drugs and/or novel delivery systems to keep the delicate balance of providing higher therapeutic effect while reducing associated side effects.

Simvastatin (SMV) is a commonly used cholesterol-lowering drug that can minimize the risk of serious cardiovascular events [2]. Recently, simvastatin has been proven to have major immunomodulatory and anti-inflammatory properties independent of cholesterol lowering [3,4,5]. These effects included improved endothelial and microvascular function, and a reduction in inflammation via reducing C-reactive protein concentrations, lowering the expression of pro-inflammatory transcriptional factors, such as nuclear factor (NF)-B, which reduces cytokines, chemokines and nitric oxide synthase (iNOS) production [6,7]. Sparrow et al. [8] have reported a potential anti-inflammatory activity of orally administered simvastatin in carrageenin-induced paw edema rat model, which was comparable to that of the standard non-steroidal anti-inflammatory drug, indomethacin. In addition, they revealed that the simvastatin-induced anti-inflammatory effect was independent of the inherent plasma cholesterol lowering effect of simvastatin. Nevertheless, despite its promising anti-inflammatory activity, the poor aqueous solubility of simvastatin might represent a major challenge against its therapeutic efficacy [9]. In addition, besides its poor solubility, SMV suffers from extensive hepatic first-pass metabolism, which significantly contributed to its limited oral bioavailability [10] and short half-life (1.5–2 h) [11]. All these factors could adversely affect the clinical efficacy of SMV following oral administration.

Transdermal drug delivery has emerged as a viable alternative to oral administration of anti-inflammatory drugs. Transdermal delivery offers several benefits over oral administration, including evading the first pass metabolism and achieving sustained release by bypassing problems associated with drug absorption through the gastrointestinal tract, such as enzyme activity and pH [12]. In addition, transdermal delivery could lower the frequency of dosing along with achieving minor fluctuations in drug plasma levels [13]. Furthermore, by being a non-invasive route of administration, the drug action may be halted quickly by removing it from the skin surface [12,14]. Consequently, transdermal administration of anti-inflammatory drugs has been widely applied for the treatment of minor strains, sprains, bruises and rheumatoid arthritis-related inflammation. Nevertheless, the stratum corneum, the outermost layer of skin, poses a permeation barrier against efficient drug absorption and, therefore, could limit the systemic bioavailability of transdermally administered drugs [15].

Recently, a growing body of literature has emphasized the potential use of lipid-based vesicles as penetration enhancers for lipophilic and/or hydrophobic drugs through the skin [16,17,18,19,20,21,22]. Lipid-based vesicles have demonstrated potential in mitigating the skin subcutaneous intercellular lipid barrier [23], allowing deep drug penetration across skin layers and its systemic absorption. Bilosomes are flexible and deformable lipid vesicles, composing of phospholipid and amphiphilic bile salts, that show superior advantages to conventional vesicular systems (liposomes and niosomes) in terms of ease of preparation, cost-effectiveness, and high stability [24,25]. In addition, the inclusion of bile salts in vesicle membrane has been reported to limit the degradation of nanovesicles in the GIT, enhance penetration and make oral delivery more effective [26]. Furthermore, bile salts significantly lower lipids phase transition temperature, making bilosomal vesicles ultra-flexible and highly deformable under physiological temperature. Such great flexibility of bilosomes enhances transdermal administration by improving penetration into the subcutaneous and skin-deep layers [26,27]. Most importantly, the inclusion of bile salts, such as sodium deoxycholate (SDC), remarkably enhances bilosomal colloidal stability, compared to other conventional vesicular systems [28]. Consequently, bilosomes have been adopted in many studies to augment the transdermal delivery of various drugs, such as ondansetron hydrochloride [29], tizanidine hydrochloride [30] and lornoxicam [31].

So far, no documented studies have assessed the potential of bilosomes as delivery vehicles for simvastatin. Accordingly, in the current study, simvastatin (SMV)-loaded bilosomal delivery system was developed and scrutinized for its anti-inflammatory potential post transdermal administration. SMV-loaded bilosomes were fabricated by thin film hydration method and optimized by Box–Behnken design. The optimized bilosomes were then characterized for particle size, entrapment efficiency and morphology. The in vitro release, ex vivo permeation, in vivo pharmacokinetics and in vivo anti-inflammatory effect of SMV-loaded bilosomes were assessed and compared with plain simvastatin.

## 2. Materials and Methods

### 2.1. Materials

Simvastatin (SMV) was kindly obtained from Hikma Pharma (Cairo, Egypt). Soybean phosphatidylcholine (SPC), sodium deoxycholate (SDC), cholesterol, Span60, hydroxypropyl methyl cellulose and dialysis bags (molecular weight cut-off of 12–14 KDa) were purchased from Sigma-Aldrich (St. Louis, MO, USA).

### 2.2. Preparation of Simvastatin-Loaded Bilosomes (SMV-BS)

The thin film hydration technique was used to prepare simvastatin-loaded bilosomes (SMV-BLs) [32]. Briefly, in a round bottom flask, SPC, cholesterol, Span 60 and SMV were dissolved in a chloroform/methanol mixture (1:1 *v*/*v*). A rotatory evaporator was employed to evaporate the organic phase at reduced pressure, resulting in the formation of a thin lipid layer. Then, the lipid film was hydrated with an adequate volume of phosphate-buffered saline (PBS, pH 7.4) containing SDC. The resultant hydrated bilosomal dispersion was finally sonicated for three cycles of 3 min at a 5 min interval to obtain bilosomes with adequate vesicle size. The sized SMV-loaded bilosomes were kept in a refrigerator at 4 °C until further use.

### 2.3. Optimization of SMV-BS

A three-factor, three-level Box–Behnken design (3^3^ BBD) was created using Design Expert software^®^ (version 12, StatEase Inc., Minneapolis, MN, USA) to scrutinize the influence of three independent variables: lipid concentration (A), SDC concentration (B) and surfactant concentration (C) on the physicochemical properties of SVM-loaded bilosomes, mainly, vesicle size (Y_1_) and entrapment efficiency (EE%; Y_2_) (Table 1). The effect of various formulation variables on the dependent variables was investigated using various experimental models, such as linear, second order and quadratic models. The regression coefficients of all models and ANOVA data were examined to select the best-fit model. The relationship between independent variables and dependent responses was then determined by the polynomial equations and 3D response plots. Finally, the point prediction approach, using desirability approach, was then applied to select the optimized formula. Under our experimental conditions, a total of 15 runs were formulated (Table 2).

### 2.4. Characterization of SMV-Loaded Bilosomes

#### 2.4.1. Particle Size and Zeta Potential

The vesicle sizes and zeta potential of SMV-BS were estimated using a Malvern particle analyzer (Nano ZS, Worcestershire, UK). After appropriate dilution with deionized water, the bilosomes were put in a disposable cuvette, then were exposed to laser diffraction for particle size and zeta potential measurement at 25 °C [33].

#### 2.4.2. Surface Morphology

SMV-loaded bilosomes morphology was examined using a Tecnai transmission electron microscopy (TEM; Philips, Eindhoven, Netherlands). The bilosomes were appropriately diluted with deionized water, put onto a carbon-coated copper grid and kept for air drying. The bilosomal sample was then inspected under a microscope at room temperature and a 50 kV acceleration voltage.

#### 2.4.3. Entrapment Efficiency

The percent entrapment efficiency (% EE) was determined by ultracentrifugation method. Briefly, bilosomal dispersion was subjected to centrifugation at 15,000 rpm for 1 h at 4 °C. The supernatant was then collected and the concentration of free unentrapped drug was quantified spectrophotometrically at λmax of 238 nm [34]. The % EE was calculated according to the following equation:

$$\%\;EE=\frac{Total\;SMV\;-\;Free\;SMV}{Total\;SMV}\times 100$$

#### 2.4.4. Differential Scanning Calorimetry (DSC) Analysis

Differential scanning calorimeter (Shimadzu, Kyoto, Japan) was used to record DSC thermograms of pure SMV, SPC, cholesterol, SDC, Span 60 and the optimized SMV-loaded bliosome formulation. In a conventional aluminum pot, 2 mg samples were heated at a constant heating rate of 10 °C/min to 300 °C at a constant heating rate of 10 °C/min under a nitrogen purge of 25 mL/min [35].

### 2.5. In Vitro Release

The drug release from optimized SMV-loaded bilosomes was compared to plain SMV suspension using a diffusion approach based on cellophane dialysis bags (MWCO 12–14 KDa). Before conducting release experiments, the dialysis bags were conditioned by soaking them in phosphate-buffered saline (PBS; pH 7.4) for 12 h. SMV-loaded bilosomes and SMV suspension (each corresponding to 10 mg SMV) were loaded in dialysis bags. The dialysis bags were placed into 900 mL release media (PBS, pH 7.4) at 37 ± 1 °C with a magnetic stirrer stirring at 100 rpm. At specified time intervals over 12 h, 3 mL aliquot samples were collected and restored with an equivalent volume of fresh media to maintain sink condition. SMV content in each sample was determined spectrophotometrically at 238 nm. The release study was conducted in triplicate and the percentage cumulative drug release was represented as a mean ± SD.

### 2.6. Physical Stability Study for the Optimized SMV-Loaded Bilosomes

The optimized SMV-loaded bilosomes were kept at 4 °C for 3 months. At 30, 60, and 90 days post-storage, bilosomes were examined for vesicle sizes and percentage entrapment efficiency.

### 2.7. Formulation of SMV-Loaded Bilosomal Gel (SMV-BS Gel)

The optimized SMV bilosomal formulation was incorporated into HPMC (2% *w*/*w*) as a gel base [36]. Briefly, a definite weight of HPMC was dispersed in a small volume of distilled water, mixed thoroughly and set aside for 4–5 h. Then, the optimized SMV-BS, containing 10 mg SMV, was centrifuged and the obtained pellets were incorporated into the gel base by a magnetic stirrer to obtain a 1% *w*/*w* smooth gel free from any aggregations. Simvastatin-loaded gel was prepared similarly but by replacing bilosomal dispersion by plain drug.

### 2.8. Characterization of SMV-Loaded Bilosomal Gel

#### 2.8.1. Physical Parameters

Visual inspection of the prepared gel was conducted to examine several physical criteria such as color, homogeneity, clarity and phase separation.

#### 2.8.2. pH Measurements

The pH of SMV-BS gel was estimated by a digital pH meter (Jenway, Staffordshire, UK). Briefly, 1 g of gel was diluted at a ratio of 1:10 with distilled water and the pH was tested in triplicate.

#### 2.8.3. Spreadability

The spreadability of SMV-loaded bilosomal gel was evaluated by placing 0.5 g of the gel in the middle between two glass slides and a fixed weight was placed over the upper slide for 1 min. The spreading area diameter was measured to determine spreadability [37].

### 2.9. Ex Vivo Permeation Study

The ex vivo permeation of SMV from SMV-BS, free SMV gel and optimized SMV-loaded bilosomal gel were tested via hairless rat skin using a Franz diffusion cell. Before beginning the experiment, abdominal rat skin was cleansed of adipose tissue and other fatty tissues, rinsed with phosphate-buffered saline (pH 7.4). Skin samples were then sandwiched between the receptor and donor compartment of diffusion cell in such a way that the stratum corneum faced the donor cell and the dermis was exposed to the receptor medium. A definite amount of SMV-BS, SMV gel or SMV-BS gel was filled in the donor compartment. The receptor compartment was filled with phosphate-buffered saline (pH 7.4) kept at 37 ± 1 °C and stirred at 100 rpm. At specified time intervals over 12 h, 2 mL aliquot samples were drawn from the receptor medium and replenished with an equal volume of fresh medium. Simvastatin concentration in each sample was assessed spectrophotometrically at 238 nm. The permeated amounts of SMV through rat skin per unit area (μg/cm^2^) were graphed versus time (h). Permeation parameters, including the flux at 24 h (J_max_) in μg/cm^2^/h, and enhancement ratio (ER) were assessed for both SMV gel and SMV-loaded bilosomal gel to verify the enhancement of SMV permeation as compared to the control.

### 2.10. In Vivo Study

#### 2.10.1. Animals

Male albino rats (220–250 g) were housed under temperature- and humidity-controlled conditions, with free access to laboratory chow and water. All animal experiments were reviewed and approved by Ethical Committee, Prince Sattam Bin Abdulaziz University, Al-Kharj, KSA (approval number: 048/2022).

#### 2.10.2. In Vivo Pharmacokinetic Study

In vivo pharmacokinetic properties of SMV from SMV gel, SMV bilosomal gel, and oral SMV suspension were compared. The rats were grouped into three groups (n = 5). The first group was orally treated with SMV suspension (10 mg/kg) via oral gavage. The other two groups were treated topically with either SMV gel or SMV bilosmal gel (10 mg SMV/kg). At specified time intervals over 24 h, 200 μL of blood was collected from the rats and centrifuged at 4000 rpm for 15 min in heparinized tubes. HPLC analysis was used to determine SMV concentration in plasma samples [37]. Briefly, Shimadzu HPLC system (Kyoto, Japan), equipped with a Thermosil^®^ C-18 column (250 mm × 4.6 mm, 5 μm) and a UV visible detector, was employed for the quantification of SMV. The mobile phase consisting of water and acetonitrile (30:70 *v*/*v*) was pumped at a rate of 1.2 mL/min. The detection was done at 238 nm, with an injection volume of 20 μL. SMV pharmacokinetic parameters, including peak plasma concentration (C_max_), half-life (t_1/2_), median residence time (MRT) and area under the plasma concentration-time curve (AUC_0-t_), were determined using a PKSolver 2.0 software.

#### 2.10.3. Anti-Inflammatory Activity

The anti-inflammatory activity of either SMV gel or SMV bilosomal gel was investigated using a carrageenan-induced rat hind paw edema test and compared with that of a standard anti-inflammatory drug, indomethacin gel. Twenty rats were divided into four groups (n = 5): Group I served as positive control; Group II: rats treated topically with 1% indomethacin gel (equivalent to 10 mg/kg indomethacin); Group III: rats treated topically with 1% SMV gel (equivalent to 10 mg/kg SMV); and Group IV: rats treated topically with 1% SMV bilosomal gel (equivalent to 10 mg/kg SMV). The paw edema was induced 30 min prior each treatment by subcutaneous injection of 100 μL of carrageenan solution (1% *w*/*v* in normal saline) in the intraplantar region of the right paw of all animals [38]. The volume of paw edema was measured before and after various treatments using a digital caliber at different time intervals over 5 h. The swelling inhibition percentage was determined using the following equation:

$$\%\;Swelling\;inhibition=\frac{V_{t}\;-\;V_{0}}{V_{0}}\times 100$$

where *V*_0_ and *V_t_* are the paw edema volume prior and after treatment, respectively.

### 2.11. Statistical Analysis

A one-way analysis of variable (ANOVA) was adopted to test the significance of differences. All values were presented as mean ± S.D. A *p*-values less than 0.05 were set as the limit of significance.

## 3. Results

### 3.1. Preparation of SMV-Loaded Bilosomes (SMV-BS)

Box–Behnken design (BBD) is a response surface approach that is widely used to produce higher order response surfaces with less necessary runs than a full factorial design. A three-level, three-factor BBD was employed to optimize SMV-BS by using distinct concentrations of lipid (SPC; A), bile salts (SDC; B) and surfactant (Span 60; C) (Table 1). The response values (vesicle size (Y_1_) and entrapment efficiency (Y_2_)) were examined for various design models, i.e., linear, second order and quadratic. Quadratic design model showed the best-fit model for the two variables. ANOVA analysis of the quadratic model for each response revealed that all the test independent variables had a statistically significant influence on all tested responses (Table S1). The predicted and adjusted R^2^ values were in reasonable agreement. In addition, the adequate precision, with a ratio greater than 4, confirmed the possibility for navigating freely throughout the design space.

### 3.2. Influence of Formulation Variables on SMV-BS Characteristics

#### 3.2.1. Impact of Formulation Variables on the Vesicle Size of SMV-BS

**Figure 1:**
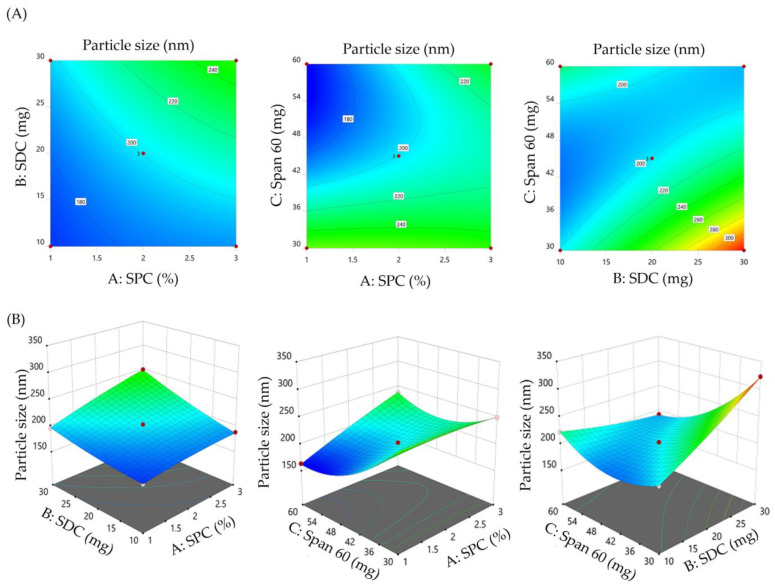
Effect of formulation variables on vesicle size (Y_1_). (**A**) Contour plots of Y_1_ and (**B**) 3D surface plots for Y_1_.

Vesicle size performs a crucial role in topical and transdermal delivery as it dictates the product pharmacologic activity. As shown in Table 2, the vesicle size of the prepared SMV-BS ranged from 163.4 ± 8.5 nm (F10) to 323.1 ± 12.9 nm (F8). Most importantly, all the tested variables exerted a significant impact on vesicle size, as depicted graphically in the contour and 3D response surface plot (Figure 1). At fixed SDC and Span 60 amounts, increasing SPC concentration from 1% to 3% resulted in a remarkable increase in bilosomal size. The mean vesicle size of F10 prepared with 1% SPC (163.4 ± 8.5 nm) was smaller than that of F6 (238.1 ± 11.7 nm). Similar findings were stated by Aldawsari et al. who emphasized the rise in vesicle size of vardenafil-loaded bilosomes upon raising the lipid concentration from 1% to 3% [32].

Similarly, vesicle size of SMV-BS was steadily increased as the SDC amount increased from 10 mg to 30 mg. Bilosomal formulation (F8), prepared with 2% SPC, 30 mg Span 60 and 30 mg SDC, showed a significantly larger vesicle size than bilosomal formulation (F3), prepared with 2% SPC, 30 mg Span 60 and 10 mg SDC. The vesicle size of F8 and F3 were 323.1 ± 12.9 nm and 201.3 ± 11.2 nm, respectively. The bile salt imparts bilosomes with a negative charge, which increase the repulsive forces among bilosomal lipid bilayers, leading to an increase in vesicle size [39]. Furthermore, the bulkiness of SDC (steroidal-like nature) could contribute to the rise in bilosomal vesicle size upon increasing SDC amount [40].

By contrary, the third independent variable, Span 60, exerted a negative effect on bilosomal vesicle size. Increasing Span 60 amount from 30 mg to 60 mg, significantly decreased the hydrodynamic diameter of SMV-BS, presumably, via reducing the interfacial tension, triggering the formation of smaller vesicles. Bilosomal formulation F10 prepared with 60 mg Span 60 had a particle size of 163.4 ± 8.5 nm, which was smaller than that of bilosomal formulation F2 (260.1 ± 12.1 nm) prepared with 30 mg Span 60.

The quadratic polynomial equation for the vesicle size is provided as follows:

Vesicle size (AY_1_) = +200.43 + 17.04 A + 22.33 B − 27.29 C + 9.55 AB + 21.22 AC − 38.05 BC + 2.90 A^2^ + 3.97 B^2^ + 30.35 C^2^ (1)

From the equation, it was evident that all formulation variables had individual and combined impacts on bilosomal vesicle size. The positive sign implies a positive effect, whereas the negative sign denotes a negative effect on vesicle size.

#### 3.2.2. Impact of Formulation Variables on the Entrapment Efficiency of SMV-BS

**Figure 2:**
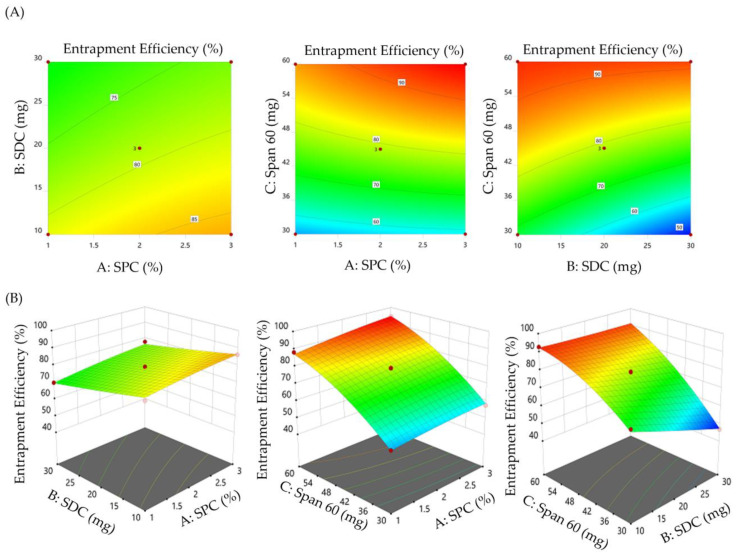
Effect of formulation variables on percentage entrapment efficiency (Y_2_). (**A**) Contour plots of Y_2_ and (**B**) 3D surface plots for Y_2_.

Generally, the development of nanocarriers with elevated drug entrapment efficiency is crucial for reducing quantity of carrier required for the administration of a definite amount of drug and minimizing drug loss during the manufacture. As summarized in Table 2, the entrapment efficiency of SMV-BS fluctuated from 46.8 ± 0.9% for F8 to 94.4 ± 4.1% for F6. In addition, ANOVA analysis revealed that all studied formulation variables significantly affect bilosomal entrapment efficiency (Figure 2). It was evident that increasing SPC concentration resulted in a mutual increase in % EE. The % EE of SMV-BS (F6) prepared at 3% SPC, 20 mg SDC and 60 mg Span 60 (94.4 ± 4.1%) was remarkably higher than that prepared with 1% SPC, 20 mg SDC and 60 mg Span 60 (88.2 ± 3.2%). Such positive effect of SPC concentration on % EE might be ascribed to the increase in lipid bilayer surface area upon raising lipid concentration, which could bestow larger space for the lipophilic drug (SMV) to be entrapped. These results are in consistence with those of Khafagy et al. who underscored the positive effect of lipid concentration on the entrapment of the antihypertensive drug, carvedilol, within bilosomes [41].

Similarly, increasing Span 60 amount from 30 mg to 60 mg resulted in an obvious rise in the entrapment efficiency. At fixed SPC and SDC concentrations, the entrapment efficiency of F10 prepared with 60 mg Span 60 was considerably higher than that of F2, formulated with 30 mg Span 60. The % EE of F10 and F2 were 88.2 ± 3.2% and 54.9 ± 1.9%, respectively. The long alkyl chain and the higher transition temperature of Span 60 might account for the higher EE of SMV within bilosomal formulations upon increasing Span 60 amount [42].

On the other hand, SDC was found to have a negative influence on the %EE of SMV-BS. Increasing SDC amount from 10 to 30 mg was found to induce a significant drop in the entrapment efficiency. The %EE of F3 prepared with 10 mg SDC was 69.2 ± 2.3%, which was significantly higher than that of F8 (46.8 ± 0.9%) prepared with 30 mg SDC. The drop in % EE might be attributed, on the one hand, to micelle formation in the dispersion media, which enhances SMV solubility and, on the other hand, to the fluidizing action of higher SDC concentrations on the lipid bilayer membrane, which leads to a decrease in % EE [43].

The quadratic polynomial equation depicting the impact of formulation variables on % EE is given below:

Entrapment efficiency (Y_2_%) = + 27.52 + 1.99 A − 2.19 B + 2.14 C + 0.04 AB + 0.06 AC + 0.03 BC − 0.65 A^2^ + 0.003 B^2^ − 0.019 C^2^ (2)

The positive sign in the equation denotes synergistic effect, whereas the negative sign denotes an antagonistic effect.

#### 3.2.3. Formulation Optimization

The optimized bilosomal formulation with desired values of dependent responses was obtained via adopting a desirability function-based numerical optimization approach. The selected optimized SMV-BS formula showed desirability value approaching to 1. The selected optimized formula, composing of 1.427% SPC, 30 mg SDC and 60 mg Span 60, showed a particle size of 172.1 ± 8.1 nm and % EE of 89.2 ± 1.8%, which were close to the predicted values (163.4 nm and 88.59%) for the optimized formulation. These results strongly emphasize the validity of 3^3^ box Behnken design for the optimization of SMV-loaded bilosomes.

### 3.3. Characterization of Optimized SMV-BS

#### 3.3.1. Particle Size, Polydispersity Index and Zeta Potential

**Figure 3:**
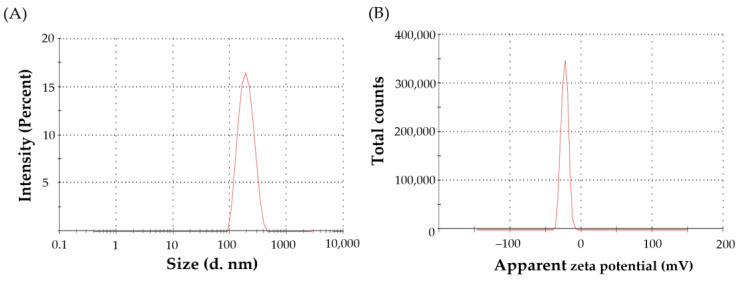
(**A**) Vesicle size and (**B**) Zeta potential of optimized SMV-BS.

The optimized SMV-BS exhibited a uniform size distribution, with an average vesicle size of 172.1 ± 8.1 nm (Figure 3A) and polydispersity index (PDI) value of 0.229, confirming the homogeneity in size distribution.

The zeta potential represents a key indicator for the storage stability and in vivo behavior of colloidal nanosystems. Generally, the electrostatic repulsion between particles imparted by high zeta potential values is crucial for minimizing the aggregation of colloidal particles [44]. In colloidal dispersion, a zeta potential of ±20 mV is required for ensuring electrostatic colloidal stability [45]. In this study, the optimized SMV-BS had a zeta potential value of −21.8 ± 2.1 mV, confirming the good physical stability of the fabricated bilosomes (Figure 3B).

#### 3.3.2. Surface Morphology

**Figure 4:**
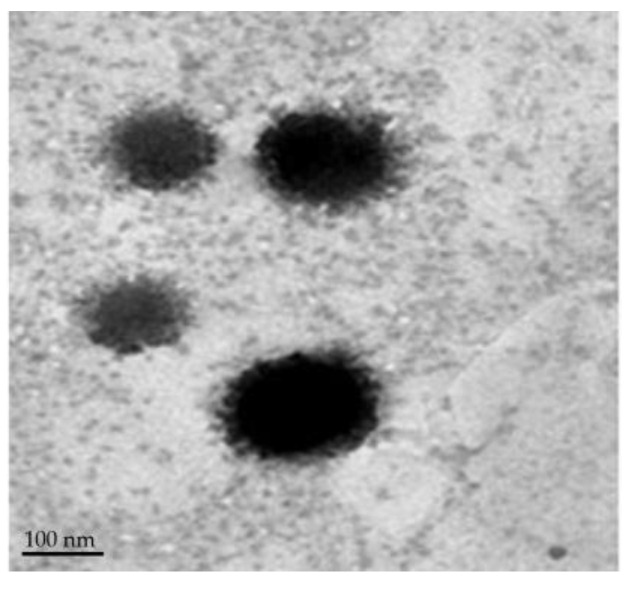
Transmission electron imaging of optimized SMV-BS.

The TEM micrographs of optimized SMV-BS (Figure 4) revealed that the prepared bilosomes were spherical in shape and having a nearly smooth surface. It also validated the particle size determined by particle size analysis.

#### 3.3.3. Differential Scanning Calorimetry (DSC) Analysis

**Figure 5:**
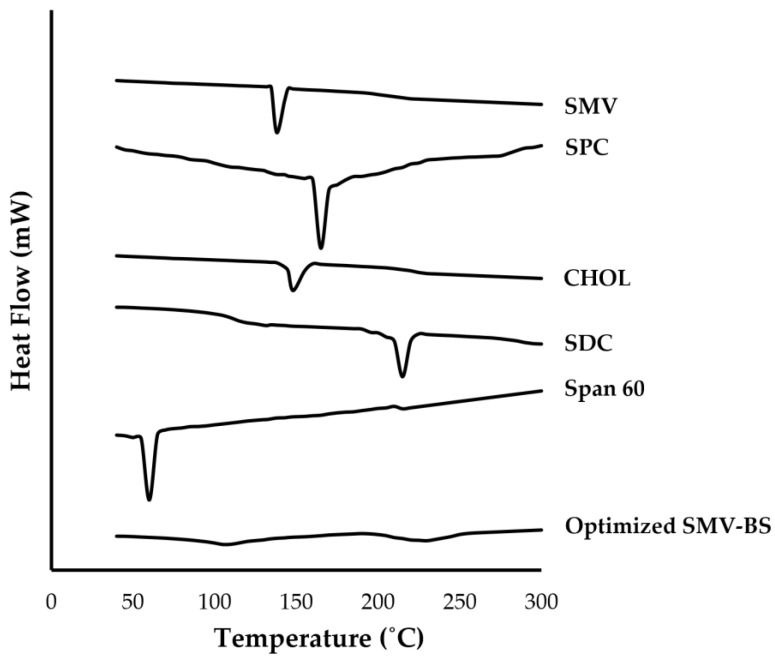
DSC thermograms of pure SMV, SPC, CHOL, SDC, Span 60 and optimized SMV-BS.

DSC analysis was conducted to assess the possible interactions between the drug and other excipients (Figure 5). According to the findings, pure simvastatin showed a strong peak at 138.4 °C, which corresponds to its melting point. The thermogram of SPC exhibited an endothermic peak at 166.9 °C. The thermograms of CHOL and Span 60 showed endothermic peaks at 148.4 °C, and 54.2 °C, respectively, representing their transition temperature. SDC thermogram showed an endothermic peak at 214 °C. Interestingly, no characteristic peaks were detected for optimized SMV-BS, suggesting that SMV molecularly dispersed within bilosomal vesicles.

### 3.4. In Vitro Release Study

**Figure 6:**
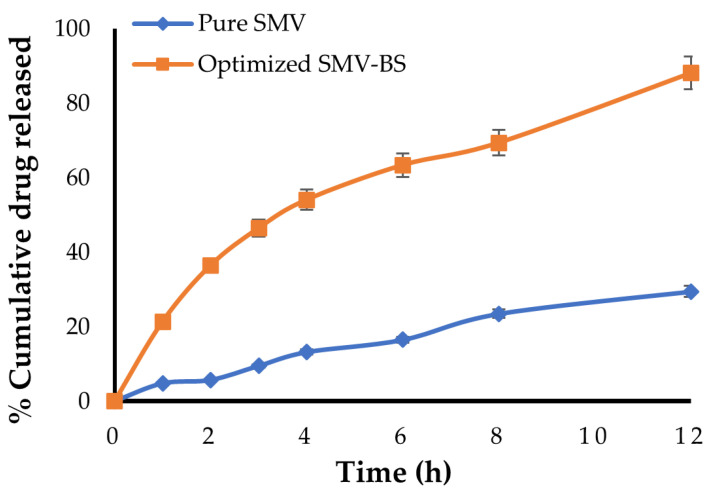
In vitro release profiles of pure SMV and optimized SMV-BS.

The in vitro release profiles of plain SMV and optimized SMV-BS were depicted in Figure 6 in terms of the percentage cumulative release of SMV as a function of time. It was clear that bilosomes potentially enhanced SMV release, compared to plain SMV suspension, with more than 85% of the encapsulated SMV released over 12 h. On the other hand, less than 35% of plain SMV was released at 12 h, presumably due to its poor aqueous solubility. In addition, SMV-BS showed a biphasic release pattern with an initial rapid drug release followed by a slower and sustained drug release for 12 h. The initial rapid drug release, where ~40% of entrapped drug was released within the first 2 h, might be ascribed to drug detachment from the bilosomal exterior surface [46]. On the other hand, owing to its high hydrophobicity, SMV tends to be detained in the lipid bilayer of bilosomes and thus exhibits slower release in the medium. This biphasic release pattern of SMV from bilosomes is anticipated to be extremely advantageous. The quick initial release of SMV, followed by a slow release over 12 h, would provide rapid drug onset while also allowing the patient to continue on therapy with fewer doses throughout the day.

### 3.5. Stability of SMV-BS

The stability of colloidal dispersions is critical concern for their successful use. In order to assess the stability of optimized SMV-BS formulation upon storage at 4 °C for 90 days, the optimized formula was monitored and analyzed at regular intervals for any changes in physical appearance and drug content. As summarized in Table 3, no evident changes in the vesicle size or drug entrapment efficiency occurred during the study period in the tested formulation.

### 3.6. Characterization of Simvastatin-Loaded Bilosomal Gel (SMV-BS Gel)

The formulated SMV-BS gel was smooth and homogeneous with no signs of phase separation. The pH of SMV-BS gel was 6.2 ± 0.15, which is considered acceptable for topical application [22] and would not cause any irritation upon application to the skin surface. In addition, SMV-BS gel showed good spreadability of 3.7 ± 0.25 cm, suggesting rapid gel spread with a small amount of shear [25].

### 3.7. Ex vivo Permeation of SMV-BS Gel through Abdominal Rat Skin

**Figure 7:**
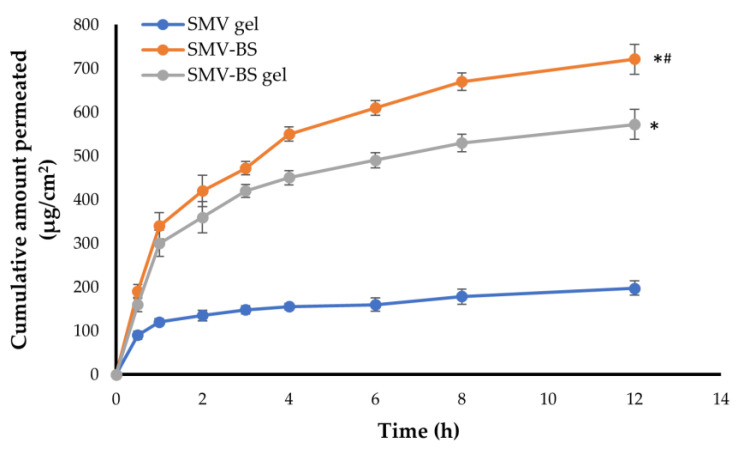
Ex vivo permeation of SMV-BS, plain SMV gel and SMV-BS gel from abdominal rat skin. * *p* < 0.05 when compared to SMV gel and ^#^ *p* < 0.05 when compared to SMV-BS gel.

Ex vivo permeation studies on SMV-BS gel as a nano-transdermal form were conducted in order to anticipate their in vivo performance. Figure 7 depicts ex vivo skin permeation of SMV-BS gel through abdominal rat skin in comparison with either SMV-BS or plain SMV gel. The developed SMV-BS gel displayed considerably greater skin penetration when compared to an equivalent amount of SMV gel (*p* < 0.05). The total amount of SMV permeated over 12 h from SMV-BS gel was 572.4 ± 34.2 μg/cm^2^, whereas only 198.1 ± 16.8 μg/cm^2^ of SMV were permeated from plain SMV gel. Furthermore, SMV-BS gel demonstrated higher flux; J_max_ of the SMV-BS gel was 47.7 ± 2.85 μg/cm^2^/h, whereas that of plain SMV gel was 16.5 ± 1.4 μg/cm^2^/h. This substantial enhancement in bilosome flux might be attributed, on the one hand, to the small vesicular size and high lipid content in SMV-BS, which promote efficient penetration of bilosomes through biological membranes [47] or, on the other hand, to the potential role of bile salts (SDC) as permeation enhancer, which could efficiently improve drug penetration by overcoming skin barrier function [42]. Of note, the ex vivo skin permeation of SMV from bilosomal gel was much lower than that from SMV-BS; the total amount of SMV permeated over 12 h from SMV-BS was 721 ± 29 μg/cm^2^, compared to 572.4 ± 34.2 μg/cm^2^ of SMV were permeated from SMV-BS gel. The relatively lower SMV permeation from SMV-BS gel might be accounted for the higher viscosity of bilosomal gel than bilosomes that could result in lower drug release from the formulation and consequently lower permeation [19]. Most importantly, transdermal diffusion of SMV from either SMV-BS or SMV-BS gel was about 3.6- and 3-fold higher than plain SMV gel, emphasizing that SMV-BS outperforms plain SMV in terms of preserving SMV release and promoting higher skin penetration.

### 3.8. In Vivo Study

#### 3.8.1. Pharmacokinetic Study

**Figure 8:**
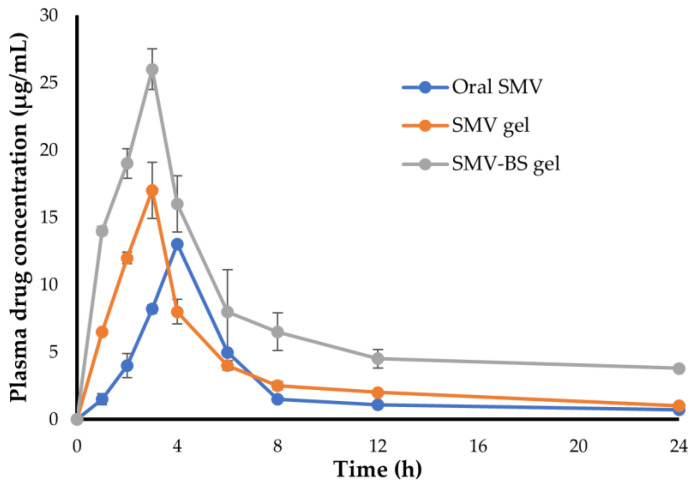
Plasma concentration vs. time curve of oral SMV suspension, topically applied palin SMV gel and topically applied SMV-BS gel. Data represent mean ± SD.

The pharmacokinetic profiles for SMV after both oral administration of SMV suspension and transdermal administration of either SMV gel or SMV-BS gel are depicted in Figure 8. As shown in Figure 8, both SMV gel and SMV-BS gel formulations exhibited higher SMV plasma concentrations, compared to oral SMV suspension. The peak plasma concentrations (C_max_) of SMV following oral administration and transdermal administration of SMV gel and SMV-BS gel were 13.20 ± 0.22 μg/mL, 17.18 ± 2.1 μg/mL and 26.32 ± 1.5 μg/mL, respectively. The relatively lower C_max_ of SMV following oral administration, compared to SMV gel formulations, might be attributed to the poor aqueous solubility of SMV, which would adversely compromise its oral absorption. On the other hand, the highest C_max_ of SMV-BS gel, compared to plain SMV gel, might be ascribed to the enhanced transdermal permeability of bilosomal formulation, which would grant efficient delivery of SMV to systemic circulation.

The key pharmacokinetic parameters of different SMV formulations were calculated and listed in Table 4. The AUC_0-24_ for SMV-BS gel formulation was found to be 177.30 ± 21.2 μg/mL.h, which was significantly (*p* < 0.05) greater than that of either plain SMV gel (85.01 ± 7.8 μg/mL.h) or oral drug suspension (60.70 ± 5.9 μg/mL.h), Moreover, SMV-BS gel significantly extended SMV residence time in the systemic circulation. The MRT of SMV-BS gel was 23.58 ± 1.7 h, which was significantly longer than those of plain SMV gel or oral SMV suspension (12.64 ± 1.0 h and 14.78 ± 1.3 h, respectively). This increase in MRT of SMV-BS gel might be related to the slow and/or prolonged release of SMV from the bilosomal vesicles and the decreased systemic clearance of SMV. Most importantly, Incorporation of SMV into bilosomal gel significantly enhanced the systemic bioavailability of SMV. The relative bioavailability of SMV-BS gel was ~2-fold and ~3-fold higher than those of oral SMV suspension and SMV gel, respectively. This increased bioavailability following transdermal administration of SMV-BS gel might be attributed to number of factors. First, bilosomal vesicles provided an efficient delivery vehicle for SMV via overcoming skin barriers. Second, bilosomal vesicles could introduce SMV in a fine molecular dispersion rather than coarse particles as in drug suspension, resulting in an enhanced surface area with a shorter diffusion route length [48]. Third, compared to oral route of administration, transdermal delivery could efficiently evade first-pass hepatic metabolism [49] and, thereby, enhance drug availability in the systemic circulation.

#### 3.8.2. Anti-Inflammatory Activity of SMV-BS Gel

**Figure 9:**
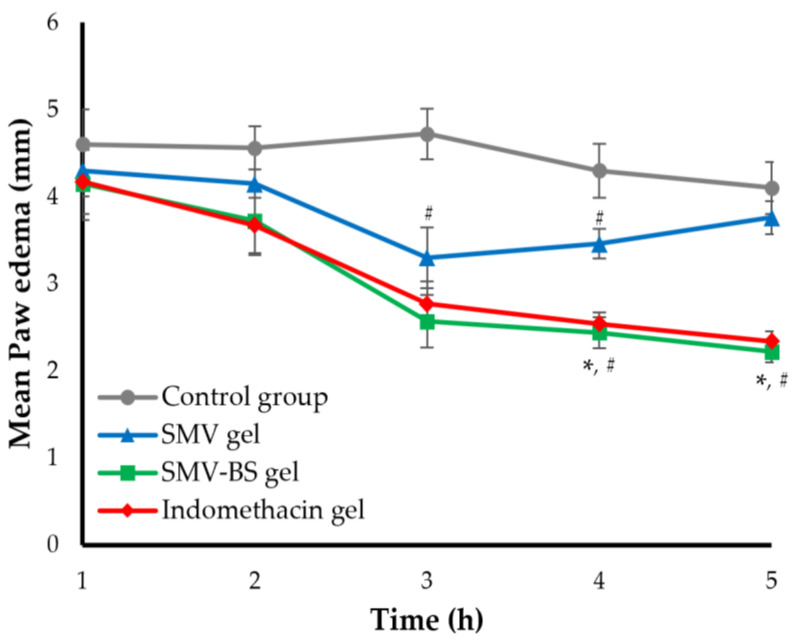
Effects of various SMV formulations on mean hind paw edema. Data are presented as mean ± SD (*n* = 5). * *p* < 0.05 compared to plain SMV gel; ^#^ *p* < 0.05 compared to control group.

The carrageenan-induced paw edema model was used to assess the anti-inflammatory potential of either SMV-BS gel or SMV gel as compared with a standard anti-inflammatory drug, indomethacin, and the inhibition of paw swelling was taken as a gauge of anti-inflammatory activity. As shown in Figure 9, subplantar injection of carrageenin (100 μL, 1% *w*/*v*) induced significant oedema in the positive control rat paws, peaking after 3 h and gradually decreasing thereafter. On the other hand, rats pretreated with either plain SMV gel, SMV-BS gel or a standard indomethacin gel exhibited a remarkable alleviation in paw edema oedema (Figure 9) when compared to control animals (*p* < 0.05). Topical application of plain SMV gel caused a significant inhibition (*p* < 0.05) in paw edema, with a maximum suppression of 30.08 ± 2.9% observed at 3 h post carrageenan administration (Table 5). Most importantly, SMV-BS gel induced a time-dependent reduction in paw edema, which was more potent than that induced by plain SMV gel (*p* < 0.5). The swelling inhibition percentages induced by SMV-BS gel were 9.78 ± 0.41%, 18.42 ± 1.1%, 45.55 ± 3.1%, 49.07 ± 3.5% and 51.95 ± 4.5% across the time period 1 h to 5 h, compared to 6.52 ± 0.32%, 8.90 ± 0.47%, 30.08 ± 2.9%, 19.53 ± 0.9 and 8.29 ± 0.81% for plain SMV gel. Such superior and prolonged anti-inflammatory effect of SMV-BS gel compared to plain SMV gel might be ascribed, on the one hand, to the enhanced transdermal permeability of SMV bilosomes and, in the other hand, to the longer MRT of bilosomal SMV, compared to plain SMV. Interestingly, the anti-inflammatory effect induced by SMV-BS gel was comparable to that of the standard anti-inflammatory drug, indomethacin (*p* > 0.05).

## 4. Conclusions

In the current study, a novel simvastatin-loaded bilosomal vesicles (SMV-BS) was introduced as a promising transdermal carrier for the treatment of inflammation. SMV-BS were prepared and optimized using Box–Behnken design. The optimized SMV-BS formula showed a nanosized range, a reasonable zeta potential, high entrapment efficiency and was efficient in sustaining in vitro drug release over 12 h. In addition, the optimized SMV-BS was then incorporated into HPMC gel. The formulated SMV-BS gel showed good physical properties and significantly showed a higher transdermal flux compared to plain SMV gel. Most notably, in vivo studies revealed that transdermal application SMV-BS gel effectively improved SMV pharmacokinetics, compared to either plain SMV gel or oral SMV suspension. In addition, in the carrageenan-induced paw edema model, SMV-BS gel elicited a potent anti-inflammatory effect, as evidenced by a notable decrease in the rat paw volume, which was comparable to that of the standard anti-inflammatory drug, indomethacin, indicating potential effects for SMV-BS gel in reducing swelling. In conclusion, bilosomes might be a plausible carrier for transdermal SMV delivery for the management of inflammation.

## Figures and Tables

**Table 1:** 3^3^ BBD design for optimization of SMV-loaded bilosomes.

Independent Variables	Code Value		
	−1	0	+1
A: SPC molar concentration	1	2	3
B: SDC amount (mg)	10	20	30
C: Span 60 amount (mg)	30	45	60
Dependent variables	Constrains		
Y_1_: Vesicle size (nm)	Minimize		
Y_2_: Entrapment efficiency (%)	Maximize		

**Table 2:** Composition of SMV-BS and their physicochemical characteristics.

Formula	Independent Variables			Responses	
	A: SPC Molar Concentration	B: SDC (mg)	C: Span 60 (mg)	Y_1_: Vesicle Size (nm)	Y_2_: Entrapment Efficiency (%)
F1	2	20	45	199.7 ± 17.3	79.1 ± 2.8
F2	1	20	30	260.1 ± 12.1	54.9 ± 1.9
F3	2	10	30	201.3 ± 11.2	69.2 ± 2.3
F4	2	10	60	222.5 ± 19.5	92.8 ± 3.1
F5	2	20	45	203.4 ± 13.4	79.3 ± 2.9
F6	3	20	60	238.1 ± 11.7	94.4 ± 4.1
F7	3	20	30	249.9 ± 17.6	57.5 ± 1.8
F8	2	30	30	323.1 ± 12.9	46.8 ± 0.9
F9	2	30	60	192.1 ± 10.8	89.9 ± 2.4
F10	1	20	60	163.4 ± 8.5	88.2 ± 3.2
F11	1	10	45	171.3 ± 11.4	79.9 ± 3.1
F12	3	30	45	250.8 ± 13.5	77.8 ± 3.6
F13	1	30	45	195.8 ± 18.6	69.9 ± 2.5
F14	2	20	45	198.2 ± 16.4	78.1 ± 1.8
F15	3	10	45	188.1 ± 15.3	86.2 ± 2.2

All data represent mean ± SD of three independent experiments.

**Table 3:** Physical stability of optimized SMV-BS.

Days Post Storage	Vesicle Size (nm)	Entrapment Efficiency (%)
Day 0	172.1 ± 8.1	89.2 ± 1.8
Day 30	175.3 ± 11.2	87.5 ± 2.5
Day 60	184.9 ± 16.7	86.9 ± 2.2
Day 90	190.1 ± 17.5	84.9 ± 3.1

Data represent mean ± SD of three independent experiments.

**Table 4:** Pharmacokinetic parameters of various SMV formulations.

Pharmacokinetic Parameter	Oral SMV Suspension	SMV-Gel	SMV-BS Gel
C_max_ (μg/mL)	13.20 ± 0.22	17.18 ± 2.1	26.23 ± 1.5
T_max_ (h)	4	3	3
T_1/2_ (h)	15.28 ± 1.1	12.08 ± 0.9	18.42 ± 1.4
K_e_ (h^−1^)	0.045 ± 0.001	0.057 ± 0.001	0.037 ± 0.001
AUC_0–24_ (μg/mL·h)	60.70 ± 5.9	85.01 ± 7.8	177.30 ± 21.2
MRT (h)	14.78 ± 1.3	12.64 ± 1.0	23.58 ± 1.7

Data presented as mean ± SD.

**Table 5:** Percentage swelling inhibition elicited by various SMV formulations.

Formula	1 h	2 h	3 h	4 h	5 h
SMV gel	6.52 ± 0.32	8.90 ± 0.47	30.08 ± 2.9	19.53 ± 0.9	8.29 ± 0.81
SMV-BS gel	9.78 ± 0.41	18.42 ± 1.1	45.55 ± 3.1	43.26 ± 3.5	45.85 ± 4.5
Indomethacin gel	9.35 ± 0.69	19.52 ± 1.7	41.31 ± 2.6	40.93 ± 1.8	42.93 ± 2.3

Data presented as mean ± SD.

## Data Availability

Not applicable.

## Supplementary Materials

The following supporting information can be downloaded at: https://www.mdpi.com/article/10.3390/polym15030677/s1, Table S1: Results of statistical analysis of all dependent variables.
