# Supplementary material for: Tailoring of Novel Bile Salt Stabilized Vesicles for Enhanced Transdermal Delivery of Simvastatin: A New Therapeutic Approach against Inflammation
Source: Polymers (Basel). 2023 Jan 29;15(3):677. doi: 10.3390/polym15030677 (PMC9921379; doi:10.3390/polym15030677)
Supplement: Supplementary file 1 [file polymers-15-00677-s001.zip › polymers-2112699-supplementary.pdf]

Table S1. Results of statistical analysis of all dependent variables

Source	Y_1		Y_2	
	F-Value	*p*-Value	F-Value	*p*-Value
Model	563.56	< 0.0001	131.09	< 0.0001
A: SPC	496.11	< 0.0001	27.66	0.0033
B: SDC	851.82	< 0.0001	99.84	0.0002
C: Span 60	1272.61	< 0.0001	979.87	< 0.0001
Lack of Fit	0.4224	0.7584	8.97	0.102
R^2 analysis				
R^2	0.9990		0.9958	
Adjusted R^2	0.9972		0.9882	
Predicted R^2	0.9925		0.9365	
Adequate precision	90.62		37.55	
